# Prevention and treatment of human papillomavirus in men benefits both men and women

**DOI:** 10.3389/fcimb.2022.1077651

**Published:** 2022-11-24

**Authors:** Kangni Zou, Yue Huang, Zhengyu Li

**Affiliations:** ^1^ Department of Gynecology and Obstetrics, West China Second University Hospital, Sichuan University, Chengdu, China; ^2^ Key Laboratory of Birth Defects and Related Diseases of Women and Children, Sichuan University, Ministry of Education, Chengdu, China

**Keywords:** couple, heterosexual, human papillomavirus, infection, sexually transmitted

## Abstract

Men should not be overlooked in research on human papillomavirus (HPV) and its associated genital diseases. This is because men infected with HPV are not only at higher risk of genital cancers, but also increase their partners’ risk of HPV infection and reinfection through sexual contact. Herein, we summarized the state of knowledge regarding the prevention and treatment of HPV infection in men as well as the possible effects of the prevention and treatment of HPV in men on their female partners. Condom use, smoking cessation, male circumcision, and HPV vaccination for men each play an important role in preventing HPV infection within heterosexual couples. Additionally, men could choose to test for certain types of HPV, such as the oncogenic HPV16 or HPV18 strains, as part of a routine screening program when their partner is positive for HPV. Although there is no recognized treatment for HPV infection as of yet, immunotherapy drugs, such as toll-like receptor agonists, therapeutic HPV vaccines, and immune checkpoint inhibitors, have shown promising results in clinical trials and in actual clinical practice. HPV infection in men also increases the risk of cervical cancer in their female partners. Because of the high partner concordance for HPV demonstrated in prior research, the prevention and treatment of HPV in men should be explored more comprehensively in future research.

## Introduction

Human papillomavirus (HPV) is the most common reproductive tract virus. HPV belongs to the Papillomaviridae family. Every year, HPV causes 630,000 cancer cases in men and women (i.e., 4.5 percent of all cancer cases), thereby posing a serious threat to public health on a global scale. In 2012, HPV-related malignancies accounted for 8.6% of all cancer cases in women and 0.8% of all cancer cases in men (Serrano et al., 2018). The Martel study, based on the data from 2012, was updated to report that cervical cancer accounted for 83% of HPV-attributable cancers, while other HPV-attributable cancers were head and neck cancer, anus cancer (half for each sex), penile cancer, vaginal cancer, and vulvar cancer (de Martel et al., 2017). Moreover, HPV is one of the most common causes of sexually transmitted diseases in sexually active women and men, causing the proliferation of scaly epithelium on the mucous membranes of human skin (Burd, 2003). At present, more than 200 HPV subtypes have been identified, of which more than 85 types have been identified in the human body (Burd, 2003). Approximately, 40 species can be transmitted to the anogenital organs and surrounding skin through sexual activity (Munoz et al., 2003).

HPV is divided into broad high-risk (hr-HPV) and low-risk types (lr-HPV) according to carcinogenicity. Infection with hr-HPV can cause head and neck cancer and oropharyngeal cancer in addition to anogenital cancer. In contrast, benign skin lesions, such as genital warts, are generally attributed to lr-HPV (Arbyn et al., 2012). Although most HPV infections and their related precancerous lesions can resolve spontaneously, HPV infection and its sequelae is still an important cause of cancers of the cervix, vulva, vagina, penis, prostate, and anus (Giuliano et al., 2015). HPV infections can also influence sperm status, potentially leading to infertility (Foresta et al., 2010). The prevention and treatment of HPV-related diseases remains a major focus in the field of medicine.

Recent studies suggest that men can take a series of measures to reduce the incidence of certain HPV types, for example, men should be vaccinated against HPV (this is especially true for adolescents) (Petrosky et al., 2015; Schmeler and Sturgis, 2016) and circumcision should be performed for males (Smith et al., 2021). However, current prevention and treatment protocols in regard to HPV infections focus only on female-specific HPV-related diseases, especially cervical cancer (which ranks fourth among female-specific diseases worldwide). There is presently sparse literature and few clinical compasses that recommend prevention, screening, and treatment for HPV infections in men.

At present, the joint prevention and treatment of HPV in men and women (which is especially critical for sexual partners) is not at the core of the screening and treatment protocols that are currently in place. However, cross-infection between couples results in persistent HPV infections more easily than in other situations, thereby elevating the risk of developing high-grade cervical lesions and eventually cervical cancer (de Lima Rocha et al., 2012). Hence, the aim of this paper is to provide an overview of the current understanding of the prevention and treatment of HPV for men and new ideas for heterosexual couples’ joint health.

## Prevention of HPV

### Condom use

Sexual contact is the primary transmission route for HPV. Males act as both virus carriers and vectors and this is an important component of the epidemiological chain for HPV (Castellsague et al., 2003). Further, women are more likely to transmit HPV to their male partners than men are to transmit HPV to their female partners (Malagon et al., 2021).

The fact that cross-infection occurs between members of a couple should not be ignored, as this is one of the reasons for the poor control of HPV evident in the literature and in clinical practice. Condoms are effective in physically isolating HPV infection, and men who do not use condoms have higher rates of HPV infection (Vardas et al., 2011). According to finding by Nielson et al. (2007), the number of condoms used in the previous three months is linked to a lower prevalence of HPV. One cross-sectional analysis covering three countries suggested that consistent condom use is an important factor in the low detection rate of any HPV type, any oncogenic type, and multiple types (Repp et al., 2012). Another cross-sectional study of 393 men showed that regular condom use during sexual intercourse is correlated with a reduction in oncogenic and overall HPV risk, which is similar to the results of the former study (Baldwin et al., 2004). Therefore, the use of condoms during sexual intercourse is essential to preventing HPV transmission and infection.

### Smoking cessation

Smoking is a known independent risk factor for HPV infection. Schabath and colleagues successively demonstrated that current smoking overall as well as current smoking with a history of greater than five pack-years of smoking was associated with a higher incidence of HPV infection (especially oncogenic infection) and a lower probability of infection clearance in men (Schabath et al., 2012; Schabath et al., 2014). Researchers have also found that smoking 10 or more cigarettes per day was associated with HPV infection in men (Nielson et al., 2007). For women, the prevalence of HPV was found to be 40.8% for smokers versus 25.2% for non-smokers; the corresponding values for men were 68.2% versus 63.2% (Kaderli et al., 2014). This suggests that men with a history of smoking are more likely to be infected with HPV than women with a history of smoking. Therefore, timely cessation of smoking in men may play an especially critical role in HPV prevention.

### Male circumcision

Numerous studies have demonstrated that male circumcision (MC) is effective in reducing the incidence of multiple HPV infection strains in men (Castellsague et al., 2002; Svare et al., 2002; Gray et al., 2010; Smith et al., 2021), thereby also decreasing the incidence of HPV-related diseases. In Baldwin and colleagues’ cross-sectional study, it was suggested that circumcision reduces the risk of overall HPV in addition to oncogenic and non-oncogenic HPV, respectively (Baldwin et al., 2004). Men who have been circumcised may be less likely to allow viral invasion through epithelial abrasions, subsequent viral shedding, and viral persistence. Thus, circumcision is also the most effective factor in reducing the clearance of oncogenic and any HPV infection (Lu et al., 2009). Similar to HPV vaccines, not only can MC help men avoid acquiring certain genital diseases, but also is beneficial to women’s health. Men who undergo MC reduce the risk of HPV infection to their female sexual partners (Morris et al., 2019). Moreover, the presence of foreskin in a woman’s sexual partner is considered a risk factor for cervical cancer (Agarwal et al., 1993). Evidence is emerging that MC can meaningfully reduce the prevalence of cervical cancer in female partners within heterosexual couples (Castellsague et al., 2002; Svare et al., 2002; Morris et al., 2019). We draw the conclusion that MC should be included as a primary preventive measure for cervical cancer, penile cancer, and other HPV-related cancers within updated medical guidelines.

### HPV vaccines

To date, 107 countries have introduced HPV vaccination programs, among which developed countries (led by Australia) have high vaccine implementation coverage; in contrast, in middle and low-income developing countries, the scale of HPV vaccine introduction has not yet been satisfactory (Bruni et al., 2021). It is thought that the HPV vaccine plays an indispensable role in preventing cervical cancer in women. However, HPV vaccination is not just an issue for women. Men are also exposed to the possibility of developing various diseases as sequalae of HPV infection. Moreover, high HPV vaccine coverage in men strongly benefits women by reducing the risk of cervical cancer (Lehtinen et al., 2018). Therefore, the HPV vaccine is undoubtedly equally needed for men and women.

A growing number of studies on gender-neutral HPV vaccination have emerged, and investigators have suggested that HPV16 eradication in the general population is predicted when 75% coverage of early adolescents (both boys and girls) is achieved (Lehtinen et al., 2019; Vanska et al., 2020). We additionally note that the herd effect refers to the indirect protective effect of vaccination on the unvaccinated population by reducing infection transmission within the susceptible population. A community-randomized trial previously reported that gender-neutral vaccination of early adolescents produced a striking population effect, substantially increasing the protective impact of vaccination on women’s health (Lehtinen et al., 2018).

Nevertheless, barriers still exist to achieving prevalent HPV vaccination in men, including but not limited to a lack of knowledge regarding HPV, prejudices against the vaccine, various sociodemographic and religious factors, fear of side effects, and concerns about cost (Grandahl and Neveus, 2021). Consequently, it is necessary to enrich knowledge regarding HPV-related diseases in the general population so that more people are willing to get vaccinated. Simultaneously, the existing healthcare system should be modified with respect to making HPV vaccines more accessible.

There are three types of HPV vaccines: a bivalent HPV vaccine, four-valent HPV vaccine, and nine-valent HPV vaccine. The bivalent vaccine protects against HPV16 and HPV18; the four-valent vaccine protects against HPV6, HPV11, HPV16, and HPV18; and the nine-valent vaccine protects against HPV6, HPV11, HPV16, HPV18, HPV31, HPV33, HPV45, HPV52, and HPV58. These three types of vaccines are all effective against HPV16 and HPV18, including HPV-related cancers, as it can be prevented effectively through the use of vaccination campaigns, as the majority of these cancers are caused by HPV16.

According to the recommendation of the Advisory Committee on Immunization Practices (Petrosky et al., 2015; Oshman and Davis, 2020), females aged 11 or 12 years should be routinely vaccinated with bivalent, four-valent, or nine-valent HPV vaccines, while males of the same age should be vaccinated with four-valent or bivalent HPV vaccine. The Advisory Committee on Immunization Practices also recommended vaccination for females and males aged 13–26 years who have not received the HPV vaccine or who have not completed the required three doses. We note that each type of vaccine is administered in a three-dose schedule. According to current recommendations, the second dose should be administered 1–2 months after the first dose and the third dose should be administered 6 months after the first dose.

## Detection of HPV

As most HPV infections clear spontaneously without intervention, a positive result does not indicate the need for immediate treatment of the patient or his or her sexual partners. Nevertheless, asymptomatic HPV infection in men is thought to be an important cause of ongoing transmission to female partners, and HPV infection in men increases the risk of cervical cancer in women (Barrasso et al., 1987). HPV infection also poses a risk for genital warts, penile cancer, and anal cancer in men. Therefore, HPV screening is necessary for men. Currently, however, only standardized HPV screening for women is emphasized, and there are no routine HPV screening programs in place for men. To our knowledge, data on the most reliable sampling site, the standardized sampling method, and the quality of sampling for men are not currently available. Some researchers suggest that, in men, samples collected from the external genital region yield a higher detection rate for oncogenic HPV than samples collected in the anal region, and the penile shaft is recommended as the optimal anatomical site for HPV detection (Nielson et al., 2007; Giuliano et al., 2007). Moreover, testing for HPV DNA appears to be the best strategy for detecting HPV infection in males, as revealed by Nicolau and colleagues. Brush material obtained from the distal urethra as well as from the external surface of the penis tends to be the most effective approach to diagnosing HPV infection in men (Nicolau et al., 2005). Targeted screening for certain types of HPV may also be used as a testing tool for HPV detection; for example, E6 seropositivity for HPV16 has been used as a prognostic and surveillance tool for oral cancer (Holzinger et al., 2017).

HPV testing for men should remain a focus within future research and clinical endeavors. We recommend several models herein: 1) men could choose to test for HPV when their female partner is positive for HPV (especially for those whose partners are positive for hr-HPV); 2) HPV screening programs for men should be developed as part of an easy and routine program; and 3) certain types of HPV infection, such as HPV16 and HPV18, should be highlighted because these infection strains are linked to the majority of malignancies of the penis, anus, and head and neck as compared with other HPV strains.

## Treatment of HPV-related lesions

Current treatment is focused on addressing individual HPV-related lesions, such as cervical cancer, vulvovaginal cancer, and penile cancer. Surgery, radiotherapy, chemotherapy, and targeted therapy are widely used in clinical practice. There is presently no standardized treatment for HPV infection only.

However, numerous studies regarding immunotherapy for HPV infection, a treatment modality that aims to achieve therapeutic goals by restoring local immune cell function, have been emerging as of now. The specific mainstream immunotherapy approaches currently under evaluation are described later in this report.

### Toll-like receptor agonists

It is well known that toll-like receptors activate innate immunity by activating downstream signaling pathways through the recognition of pathogen associated molecular patterns, thereby stimulating the production of proinflammatory cytokines and type I interferons (Mifsud et al., 2014; Owen et al., 2020). In summary, toll-like receptor agonists stimulate the body’s innate immune system and enhance innate immune function to clear pathogens and protect the body from infection (Mifsud et al., 2014). Imiquimod, a typical toll-like receptor agonist, has been increasingly used in the treatment of HPV-associated intraepithelial neoplasia and squamous cell carcinoma *in situ* of the penis as an alternative treatment option to surgery, with fewer adverse events and tumor recurrence (Schroeder and Sengelmann, 2002; Tristram et al., 2014).

### Therapeutic HPV vaccines

Among the HPV proteins, E6 and E7 (proteins involved in tumorigenesis and progression) are considered ideal targets for cervical cancer immunotherapy (Pal and Kundu, 2019). Therapeutic HPV vaccines targeting E6 and E7 proteins have therefore been proposed; these vaccines are capable of enhancing the T cell immune response (Garbuglia et al., 2020). Live-vector-based, peptide-based, protein-based, dendritic cell-based, and genetic vaccines each have great advantages as well as demonstrated effectiveness in the treatment of HPV-related diseases (Chandra et al., 2021). Although no vaccine has presently been approved for clinical use, the therapeutic HPV vaccine has a promising future as an effective treatment strategy.

### Immune checkpoint inhibitors

Immune checkpoints and their ligands have been found to be constantly upregulated in the tumor microenvironment of diverse malignancies, representing a major obstacle in the initiation of the body’s effective innate anti-tumor immune response (Toor et al., 2020). Immune checkpoints inhibitors (ICIs) have become a popular research target in the field of tumor immunotherapy and are currently the main therapeutic strategy employed in immunotherapy. ICIs induce the blockade of programmed cell death protein 1, programmed death-ligand 1, and cytotoxic T-lymphocyte-associated protein 4. Ipilimumab (targeting cytotoxic T-lymphocyte-associated protein 4), and nivolumab and pembrolizumab (both targeting programmed cell death protein 1) are presently licensed for marketing. Moreover, programmed cell death protein 1 inhibitors have entered clinical trials with respect to the treatment of advanced cervical cancer following HPV infection (Chung et al., 2019; Naumann et al., 2019). Still, the clinical efficacy of any particular ICI alone is limited; according to current findings, ICIs should instead be combined with other therapeutic modalities to improve treatment outcomes. In summary, more convincing clinical trials proving the efficacy of this treatment modality are needed to address the current difficulties in the application of immunotherapy in HPV-related diseases.

## Necessity of HPV treatment for men

Reciprocal transmission of HPV is prevalent between couples. In a previous study conducted by Burchell and colleagues that evaluated couples in which both partners were positive for any type of HPV, 87% were found to be concordant for at least one type of HPV strain (Burchell et al., 2010). Compelling evidence from a well-designed cross-sectional study conducted in 2014 showed that 68% of the evaluated couples in which both partners were positive for HPV had at least one genotype (i.e., strain) in common; moreover, if male partners were positive for at least one HPV genotype, this had a substantial impact on their female partners’ positivity status (de Lima Rocha et al., 2012). Moreover, Bleeker et al. demonstrated a high concordance of partner HPV types, and presented findings that this concordance may be associated with an increased viral load (Bleeker et al., 2005).

Among heterosexual partners, female patients diagnosed with cervical intraepithelial neoplasia have been shown to increase the risk of HPV infection in their sexual partners (Martin-Ezquerra et al., 2012). Although the majority of infections are cleared through enhanced immune function, there is still a risk of progression to severe disease among those infected with HPV.

## Discussion

Men are an important component of the cycle of the transmission of sexually transmitted diseases, and that HPV-positive men are also responsible for their female partners’ reinfection status (Skoulakis et al., 2019). As carriers and vectors of hr-HPVs, male partners may also cause a significant impact on the development of cervical cancer in their female partners (Skegg et al., 1982; Barrasso et al., 1987; Bosch et al., 1996). This reminds us that HPV-specific treatment and preventive medicine efforts should not only be targeted to women, but also toward men with HPV infections.

Hence, in addition to various screening and preventive efforts, the co-treatment of HPV for male and female partners is extremely crucial to slow down HPV transmission. Moreover, partner co-therapy for HPV infection should gradually be included within clinical treatment practices and formal medical guidelines informing clinical decision-making.

## Conclusion

HPV infection in men and the effects of HPV infection on their partners are increasingly being emphasized in the medical literature, in ongoing epidemiologic and clinical research efforts, and in clinical practice. Since sexual transmission is the main route of HPV infection, condoms can help prevent HPV transmission by physically isolating contact with the mucous membranes of the skin. Smoking has also been recognized as an independent factor contributing to HPV infection, and quitting smoking is one of the known measures for preventing HPV. Moreover, studies have successively proven that MC and male vaccination exert a crucial effect on preventing HPV in female sexual partners.

We also note that, on the one hand, HPV infection in men may be a potential risk factor for cervical cancer in women. On the other hand, the high concordance of HPV types in partners suggests that cross-infection is a barrier to HPV control. Therefore, in summary, the treatment of HPV infection in men should undoubtedly be addressed equally as that for women.

Early intervention in men can protect against the transmission of HPV infection, while also reducing the incidence of cervical cancer in female partners and facilitating the treatment of female patients with HPV. However, there is still much to learn about the prevention and treatment of HPV and its related malignant diseases. We strongly recommend that this disparity be investigated in male patients as well in female patients, and that this should become a key focus and hotspot for future research.

## Author contributions

KZ contributed to the manuscript drafting and final approval. YH contributed to manuscript revising and critical discussion. ZL provided practical suggestions and critically revised the manuscript. All authors have read and approved the final manuscript. All authors contributed to the article and approved the submitted version.

## Funding

This study was supported by the Medical Science and Technology Project of Sichuan Provincial Health Commission (No. 21PJ050).

## Acknowledgments

We acknowledge the staff of the Key Laboratory of Birth Defects and Related Diseases of Women and Children (Sichuan University), Ministry of Education, Chengdu, People’s Republic of China, for the methods instruction of the study.

## Conflict of interest

The authors declare that the research was conducted in the absence of any commercial or financial relationships that could be construed as a potential conflict of interest.

## Publisher’s note

All claims expressed in this article are solely those of the authors and do not necessarily represent those of their affiliated organizations, or those of the publisher, the editors and the reviewers. Any product that may be evaluated in this article, or claim that may be made by its manufacturer, is not guaranteed or endorsed by the publisher.

## Abbreviations

HPV, human papillomavirus; hr-HPV, high-risk human papillomavirus; lr-HPV, low-risk human papillomavirus; MC, male circumcision; ICI, immune checkpoints inhibitor.
